# Breast cancer suspected to originate from familial hereditary tumors: A case report

**DOI:** 10.1002/ccr3.2698

**Published:** 2020-02-08

**Authors:** Shinya Yamamoto, Takashi Chishima, Sadatoshi Sugae, Yukako Shibata, Akimitsu Yamada

**Affiliations:** ^1^ Department of Breast Surgery Fujisawa City Hospital Fujisawa City Kanagawa Japan; ^2^ Department of Breast Surgery Yokohama Rosai Hospital Yokohama city Kanagawa Japan; ^3^ Department of Breast Surgery Chigasaki Municipal Hospital Chigasaki City Kanagawa Japan

**Keywords:** breast cancer, genetic testing, hereditary tumor, pancreatic tumor

## Abstract

If familial hereditary tumor is suspected, diagnosis and treatment should always be performed considering the presence of familial hereditary tumors irrespective of whether genetic testing is performed.

## Graphical abstract

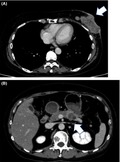

## 1 INTRODUCTION

A 51‐year‐old woman presented with locally advanced stage 4 breast cancer and a pancreatic tumor. Although the breast tumor shrank after 4 courses of chemotherapy, the pancreatic tumor progressed and became unresectable; biopsy confirmed it to be a primary pancreatic carcinoma. She is currently undergoing chemotherapy for pancreatic carcinoma.

Breast cancer is the most frequently diagnosed cancer in women and ranks second among the causes of cancer‐related death in women. Reports suggest that 7%‐10% of breast cancers are caused by mutations in specific genes. The guidelines also indicate the importance of identifying hereditary breast cancer.

Multiple tumors are often observed in the same patient, and a hereditary tumor is occasionally suspected. The analysis of hereditary tumors has advanced in recent years, and abnormal genes have been identified. Breast cancer care requires attention to the presence of hereditary tumors. Here, we present a case that suggested the possibility of a familial hereditary tumor.

## 2 CASE HISTORY/EXAMINATION

**Figure 1:**
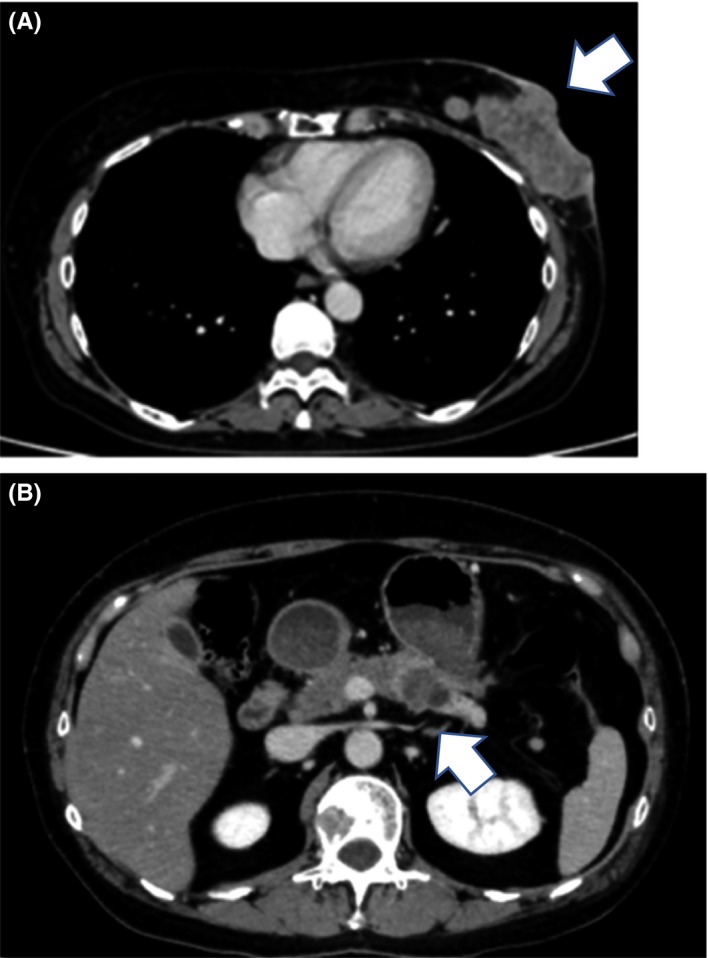
Computed tomography before treatment. A, A mass measuring 7 cm in the left breast with ulceration (Arrow). B, A mass measuring 2 cm in the tail of the pancreas (Arrow)

**Figure 2:**
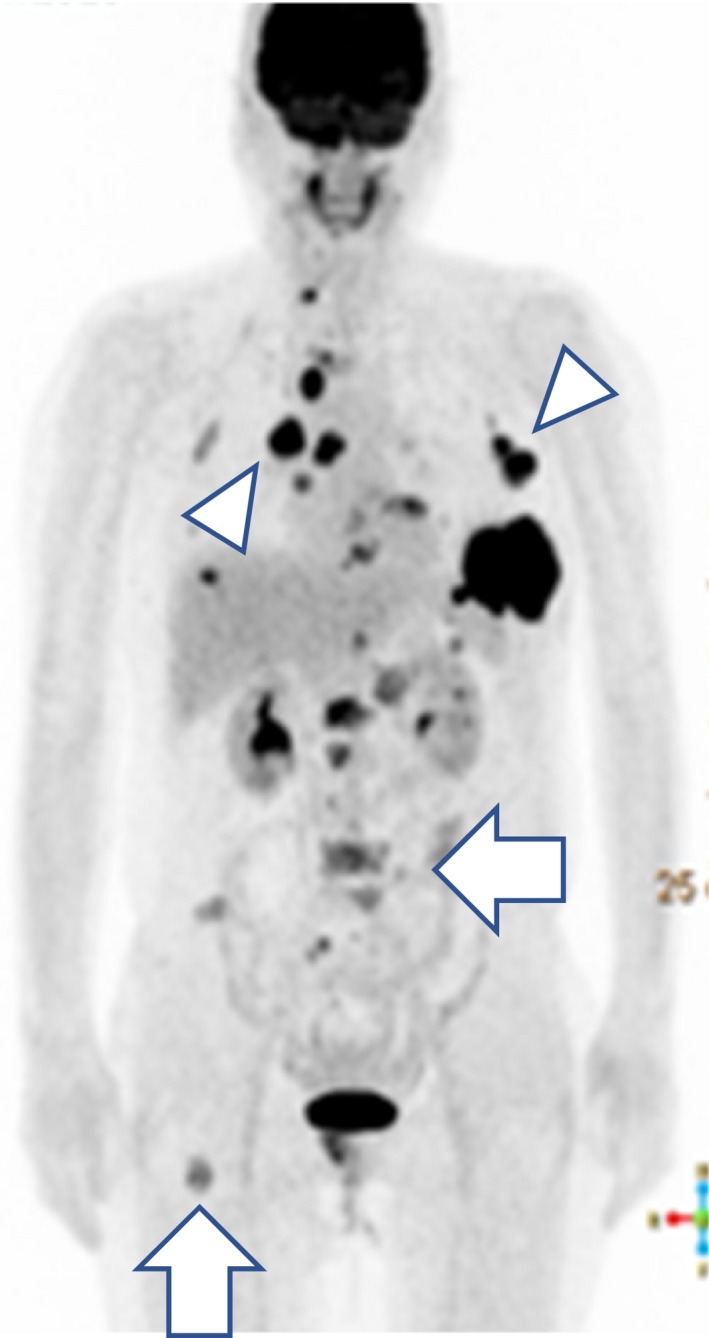
Positron emission tomography before treatment. Accumulation detected in numerous lymph nodes (Arrow head) and bones including the spine and right femur (Arrow)

A 51‐year‐old woman visited our hospital owing to a mass in her left breast. Ulceration was observed in the mass with enlarged lymph nodes in the axilla. Computed tomography (CT) revealed a mass in the left breast measuring 7 cm and axillary lymph node metastases (Figure 1A). Core needle biopsy provided a diagnosis of breast cancer, and fine needle aspiration biopsy diagnosed the lymph node metastases. CT also revealed a pancreatic tumor, (Figure 1B) while positron emission tomography (PET) revealed a bone tumor (Figure 2). Primary pancreatic tumors were considered, with metastasis to the left breast; however, till this point, the bone tumor was diagnosed as metastasis from breast cancer. A mass measuring 1.5 cm was observed in the right breast; it was diagnosed as either a second primary tumor or metastasis from the left breast tumor. Several lymph node and lung metastases were observed. Therefore, the left breast cancer was staged as T4bN1M1, cStage4. Immunostaining of the left breast tumor revealed estrogen receptor (ER) and progesterone receptor (PgR) positivity, human epidermal growth factor receptor (HER2) negativity, and a Ki‐67 labeling index of 30%. The tumor marker levels were as follows: CEA: 11.4 ng/mL, CA15‐3:40.6 U/mL, and CA19‐9:3.3 U/mL.

The patient provided a history of childhood leukemia and her family history suggested that her father had gastric cancer, colon cancer, and lung cancer. Her paternal grandfather and grandmother had gastric cancer. One of her paternal uncles had gastric cancer, laryngeal cancer, and myelodysplastic syndrome, while another had gastric cancer, lung cancer, and osteosarcoma; a third relative had pancreatic cancer. We therefore recommended genetic testing, which the patient refused.

Scheduled chemotherapy was initiated for breast cancer as it was locally advanced. Additionally, the scheduled chemotherapy was considered to be effective for treating primary pancreatic tumors.

**Figure 3:**
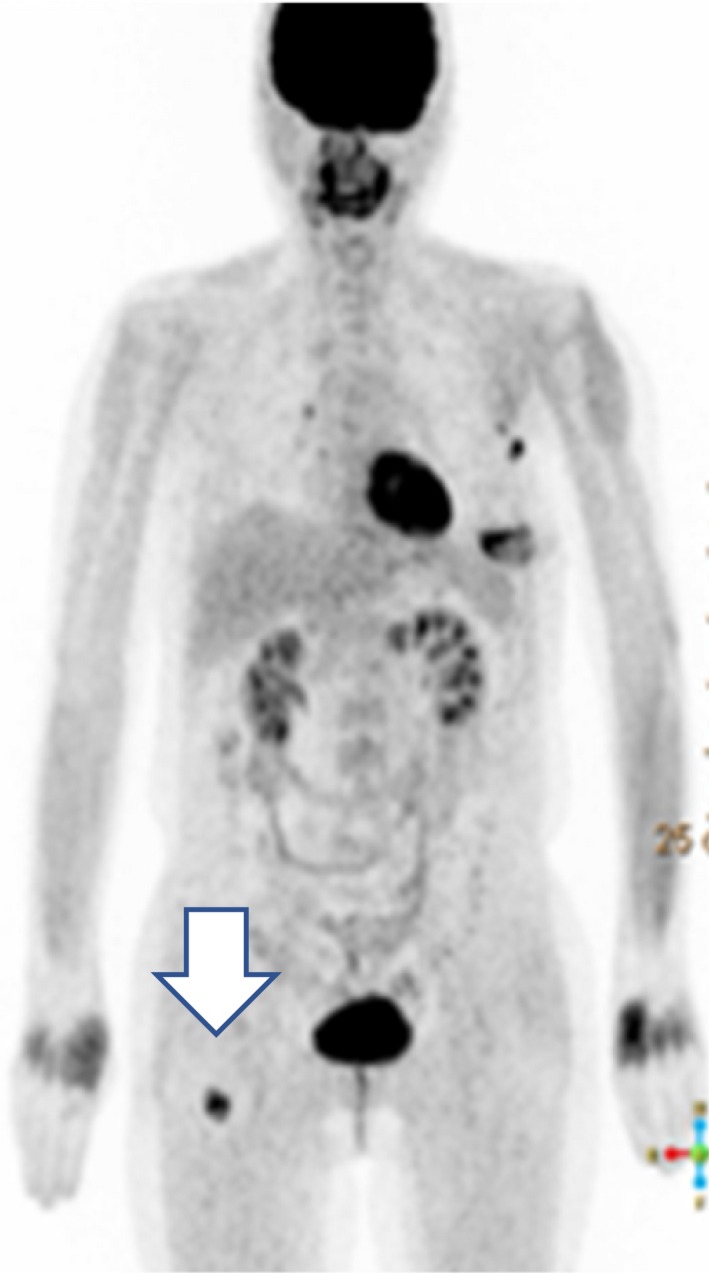
Positron emission tomography after 2 cycles of paclitaxel + bevacizumab. Disappearance of bone metastases with accumulation in the right femur indicating the presence of a tumor (Arrow)

The regimen comprised paclitaxel (90 mg/m^2^, days 1, 8, and 15) and bevacizumab (10 mg/m^2^, days 1 and 15), administered every 28 days. Zoledronate (4 mg) was administered every 4 weeks. After 2 courses of paclitaxel and bevacizumab therapy, she developed influenza and experienced severe numbness in her fingers due to paclitaxel. Chemotherapy was therefore temporarily withheld. We biopsied the pancreatic tumor in the interim; it was found to be an adenocarcinoma. Determination of the primary tumor site was challenging due to an insufficient sample volume. CT and PET revealed shrinkage of the breast tumor, lymph node metastases, and the pancreatic tumor. Except for that in her right femur, the bone metastases had nearly disappeared (Figure 3). Chemotherapy was deemed effective, and after recovery from influenza and the numbness in her finger, chemotherapy was continued at 80% of the original dose. At this time, the bone tumors were considered to be osteosarcomas. After consultation with orthopedists, we decided not to treat the bone tumors as she had no fractures or pain. We recommended genetic testing again; however, she refused.

**Figure 4:**
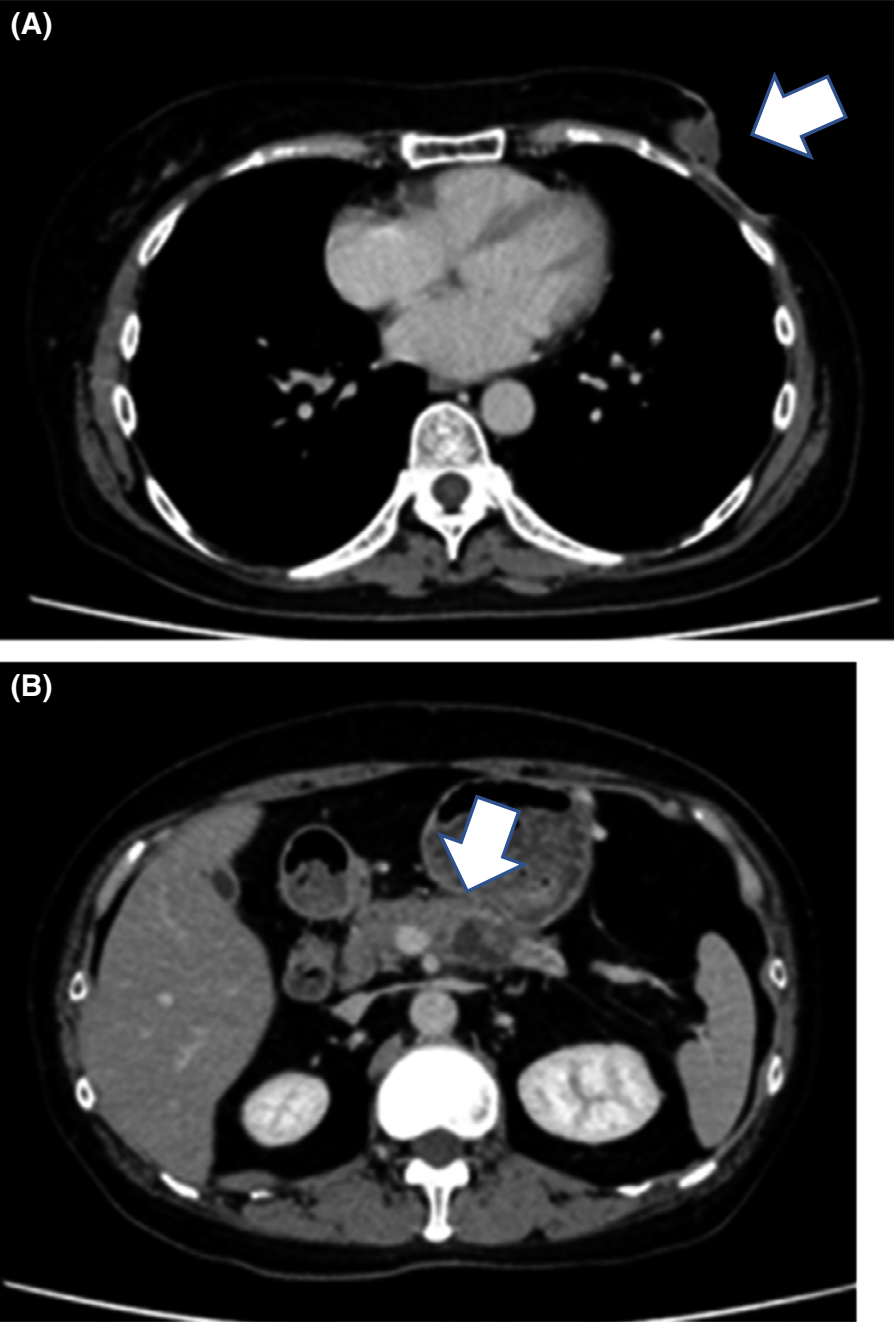
Computed tomography after 4 cycles of paclitaxel + bevacizumab. A, Shrinkage of the tumor in the left breast (Arrow). B, Increase in the size of pancreatic tumor, which is seen infiltrating the superior mesenteric artery (Arrow)

CT was performed following 2 further courses of chemotherapy. Although the breast tumor remained unaltered (Figure 4A), the pancreatic tumor had increased in size (Figure 4B); on rebiopsy, it was identified to be a primary pancreatic ductal carcinoma. Pancreatic tumor cells showed mucus production, which was confirmed by Periodic acid‐Schiff (PAS) and Alcian blue staining. Immunostaining demonstrated GATA3, ER, and PgR negativity. The pancreatic tumor was therefore diagnosed to be a pancreatic duct carcinoma.

The pancreatic tumor had infiltrated the superior mesenteric artery and was deemed unresectable. Since the pancreatic cancer was the predominant prognostic factor at this stage, FOLFIRINOX (leucovorin and fluorouracil plus irinotecan and oxaliplatin) chemotherapy was initiated; the patient is currently undergoing this treatment.

## 3 DISCUSSION

In this case, the patient was diagnosed with locally advanced tumors, which were believed to originate from a familial hereditary tumor. The patient suffered from multiple metachronous and synchronous malignancies, and several members of her paternal lineage had cancer. Genetic testing was recommended, but not desired; therefore, her final diagnosis remains unconfirmed.

In this case, we suspected Li‐Fraumeni syndrome (LFS) or hereditary breast and ovarian cancer (HBOC). LFS is a familial genetic syndrome that causes multiple cancers and is transmitted via autosomal dominant inheritance. LFS is suggested to be associated with germline mutations of the TP53 tumor suppressor gene.^1^ LFS was initially described in 1969 by Frederick Li and Joseph Fraumeni in four families.^2^ There are fewer than 400 family reports worldwide. LFS patients are generally at an increased risk of developing cancer at a younger age: 50% by 30 years and >90% by 60 years.^3^ LFS patients are also at a high risk of developing multiple primary cancers, with a 57% and 38% risk of developing 2 and 3 types of cancer, respectively. Primary cancers include soft tissue sarcomas, osteosarcomas, premenopausal breast cancer, brain tumors, and adrenal cortex carcinomas; these account for approximately 80% of all tumors associated with LFS. In addition, several studies report that in these patients, leukemia, bronchoalveolar carcinoma, colon cancer, skin cancer, stomach cancer, and ovarian cancer are each diagnosed at a younger age than the average age of onset.^4^, ^5^ In our case, the patient was diagnosed with breast cancer at the age of 51 years; however, the breast cancer was advanced, and the onset could have occurred several years previously.

HBOC patients have a mutation in one or both of the *BRCA1* and *BRCA2* genes, which function to repair DNA damage. These patients are prone to breast or ovarian cancer.^6^ Reports suggest that 7%‐10% of breast cancers are caused by mutations in specific genes. A significant proportion of these are attributable to mutations in the *BRCA1* and *BRCA2* genes. In cases where there is a mutation in either *BRCA1* or *BRCA2*, the cumulative risk of developing breast cancer by the age of 80 years is approximately 70%; when there is a mutation in *BRCA1*, the risk of developing ovarian cancer is 44%, and when there is a mutation in *BRCA2*, the risk is 17%.^7^ Patients with HBOC are also prone to prostate (males) and pancreatic cancer.^6^ In our case, although there were no breast cancer patients in the family, the number of women was low; the possibility of HBOC could not be excluded.

It is possible that the bone tumor was related to metastatic breast cancer and osteosarcomas. This was indicated by the fact that chemotherapy was effective in treating the other bone metastases but not the right femoral tumor. If the right femoral tumor would be identified as osteosarcoma, the patient would have been diagnosed with four cancers.

Yamashita et al reported on the utility of immunohistochemical staining for MUC5AC in differentiating primary pancreatic cancer from pancreatic metastasis of breast cancer.^8^ Although our case was similar to the case described by Yamashita et al, we did not use this method. We diagnosed pancreatic cancer owing to the presence of mucus production and, ER, PgR and GATA3 negativity. ER and PgR positivity was observed in the primary breast cancer site, whereas the pancreatic tumor tested negative. Since the sensitivity of GATA3 for metastatic breast cancer ranges from 73% to 96%,^8^ we considered the pancreatic tumor not to be a lesion of metastatic breast cancer. However, Yamashita et al also reported that the range of sensitivity does not adequately exclude metastatic breast cancer in GATA3‐negative pancreatic tumors. Unlike the case described by them, our case was judged to involve a primary pancreatic tumor as the histological features of hematoxylin and eosin (HE) staining differed between the primary lesion and pancreatic tumor.

The recommended treatment for resectable pancreatic cancer is surgery. Chemotherapy such as gemcitabine, S‐1, FOLFIRINOX, and gemcitabine plus nab‐paclitaxel is recommended in unresectable cases.^9^ Reports confirm the utility of preoperative chemotherapy for resectable pancreatic cancer^10^ and that of paclitaxel plus bevacizumab in the first‐line treatment of HER2‐negative metastatic breast cancer.^11^

In this case, a primary pancreatic cancer was diagnosed in addition to advanced breast cancer. However, the breast cancer was treated first owing to the advanced stage at presentation. The planned chemotherapy was expected to be effective for pancreatic cancer, and there was no time for diagnosing the pancreatic tumor.

The pancreatic tumor was biopsied when the chemotherapy was suspended due to its side effects. However, a definitive diagnosis was not possible in the absence of specimens. Nevertheless, treatment was continued as both the breast and pancreatic tumors appeared to be shrinking on imaging. Subsequent examination diagnosed the primary pancreatic cancer. Since primary pancreatic cancers are treated by resection if resectable, the course of treatment may have been different if the primary pancreatic cancer was diagnosed at the first visit. However, in the event of peritoneal dissemination, resectability would be affected. In addition, pancreatectomy is a difficult operation with the possibility of postoperative complications, and subsequent treatment delays. That may have resulted in a poor prognosis. Furthermore, although the patient was initially diagnosed with resectable pancreatic cancer, upfront administration of preoperative chemotherapy was possible; the treatment method may have therefore been altered.

In the case of multiple tumors, numerous examinations are required; this requires considerable time to complete all diagnoses. Therefore, it is sometimes necessary to start treatment before completing diagnosis. However, it is necessary to proceed with treatments and examinations considering the paucity of diagnoses. Various tumors in the same patient are often considered metastases from one primary lesion; however, the possibility of multiple primary tumors must always be considered, particularly in case of hereditary tumors. In this case, if we were more aware of the presence of a hereditary tumor, we may have strongly considered the pancreatic tumor to be a primary tumor. Imaging and pathology were important for diagnosis; however, genetic understanding of the tumors is also essential.

The significance of genetic testing must be emphasized for the patient to make informed decisions regarding their treatment, surveillance for other tumors, and surveillance for family members. In particular, LFS patients must avoid radiation exposure,^12^ and patients with HBOC should consider undergoing preventive mastectomy and preventive ovariectomy.^13^ If a familial hereditary tumor is suspected, it is necessary to suggest that a genetic test be taken; however, even if refused, the relatives should be counseled accordingly. Certain relatives including living siblings will benefit from genetic testing. However, in this case, there the opportunity for interviews with relatives was scarce, and we were not able to propose it.

## 4 CONCLUSION

Knowledge of hereditary tumors has particular significance in terms of diagnosis, irrespective of whether genetic testing is performed.

## CONFLICT OF INTEREST

The authors declare that there are no conflicts of interests regarding the publication of this article.

## AUTHOR CONTRIBUTIONS

SY: acquired the data, analyzed and interpreted the data, wrote the manuscript, and approved the final manuscript. TC: contributed to patient evaluation and approved the final manuscript. SS, YS, and AY: analyzed and interpreted the data, approved the final manuscript.
